# Frequent Eating Out and 10-Year Cardiovascular Disease Risk: Evidence from a Community Observatory in Malaysia

**DOI:** 10.1155/2022/2748382

**Published:** 2022-03-07

**Authors:** Chiew Way Ang, Roshidi Ismail, Zaid Kassim, Ahmad Nizal Mohd Ghazali, Daniel Reidpath, Tin Tin Su

**Affiliations:** ^1^South East Asia Community Observatory (SEACO) & Global Public Health Jeffrey Cheah School of Medicine and Health Sciences, Monash University Malaysia, 47500 Subang Jaya, Selangor, Malaysia; ^2^Segamat District Public Health Office, Ministry of Health, 85000 Segamat, Johor, Malaysia; ^3^International Centre for Diarrhoeal Disease Research, Bangladesh

## Abstract

Despite increasing mortality rates from cardiovascular diseases (CVDs) in low- and middle-income countries, information on the estimation of 10-year CVD risk remains to be sparse. Therefore, this study was aimed at predicting the 10-year CVD risk among community dwellers in Malaysia and at identifying the association of distal (socioeconomic characteristics) and proximal (lifestyle practices) factors with 10-year CVD risk. We calculated the 10-year CVD risk score among 11,897 eligible respondents from the community health survey conducted by the South East Asia Community Observatory (SEACO) using the Framingham risk score (FRS). The findings indicate that 28% of respondents have a high chance of having CVD within the next ten years. After adjusting for the age of respondents, demographic and socioeconomic factors such as gender, ethnicity, marital status, education, income, and occupation had an association with the 10-year CVD risk. In addition, frequent eating out had an association with 10-year CVD risk, while physical activity was found to have no association with predicted CVD risk. CVD remained among the top five mortality causes in Malaysia. Health promotion strategies should emphasize the importance of having home-cooked meals as a healthy dietary behavior, to reduce the mortality rate among Malaysians due to CVDs.

## 1. Introduction

Cardiovascular diseases (CVDs) account for the majority of noncommunicable disease (NCD) deaths across the globe [1]. NCDs contributed to about 73% of the total deaths in Malaysia, with the largest contributor being CVDs (35%) in 2015 [2]. Ischaemic heart disease remained among the five principal causes of deaths in Malaysia (15.6%) in 2019 [3].

In addition, the prevalence of obesity (17.7%), hypercholesterolemia (47.7%), and diabetes (17.5%) had increased among Malaysian adults [4]. Since individuals may have one or more CVD risk factors and/or chronic disease conditions, the progression of a particular CVD risk factor cannot accurately predict future CVD risk at the population level. The Framingham risk scoring (FRS) model can predict the CVD outcomes “fairly well” in the Asian population [5–9]. However, there remain to be limited findings on the 10-year CVD risk predicted by using a representative population sample in Asian countries with developing economies such as Malaysia.

In the related literature, the association between behavioural or lifestyle exposure such as unhealthy diets and physical inactivity with individual CVD risk factors such as obesity [10–12], hypertension [13, 14], diabetes [11], and hyperlipidaemia [14, 15] has been documented. However, past studies investigating the relationship between lifestyle factors such as diet and physical activity with 10-year CVD risk are lacking. Hence, this study was aimed at predicting the 10-year CVD risk among Malaysians and at identifying the association of distal (socioeconomic characteristics) and proximal factors (lifestyle practices) with a 10-year CVD risk.

## 2. Materials and Methods

### 2.1. Study Design and Data Collection

This study utilized data from the community health survey 2013 collected by the South East Asia Community Observatory (SEACO). SEACO is a health and demographic surveillance system (HDSS) established in Segamat, Johor, Malaysia [16]. This surveillance site covers approximately 44,900 individuals that reside in 13,400 households in the baseline enumeration (household census) conducted in 2012 [16]. SEACO conducted house-to-house interviews to obtain the demographic and socioeconomic status (e.g., education, age, ethnicity, and income) and self-reported information on lifestyle practice and dietary behaviour (e.g., smoking, physical activity, and frequency of eating outside). Anthropometric measurements (e.g., height, weight, blood pressure, and random blood glucose) were also conducted as part of the home-based health screening among respondents aged 35 years and above. Data collection was undertaken from August 2013 to July 2014. The total number of respondents in this survey was 25,184 [16].

### 2.2. Selection of Respondents

**Figure 1:**
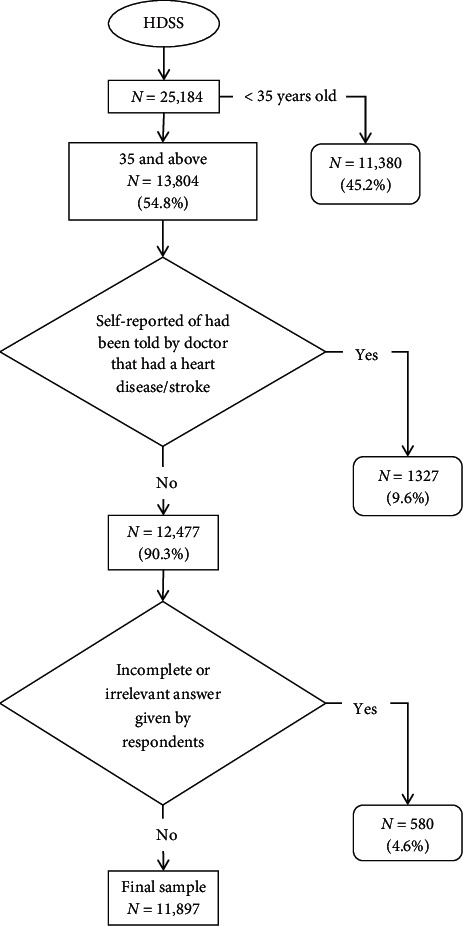
Study sample.

The respondents with no history of CVD were eligible for this FRS prediction model. From among 25,184 respondents, only 13,804 had blood pressure measurement. In addition, we considered the exclusion of the respondents that answered “Yes” to the following questions: (1) “Have you ever been told by a doctor/medical assistant that you have heart disease?” or (2) “Have you ever been told by a doctor/medical assistant that you have had a stroke?”. However, the number of respondents was further reduced to 11,897 because of missing information (Figure 1). We included all eligible participants in our data analysis.

### 2.3. Cardiovascular (CVD) Risk Score

The outcome variable was modified FRS point proposed by D'Agostino et.al [17] to determine the 10-year CVD risk. To estimate the 10-year CVD risk score, the researchers utilized nonlaboratory predictors: age (in years), body mass index (BMI), antihypertension medication use, systolic blood pressure (SBP), smoking status, and diabetes mellitus status. Respondents were classified as smokers if they reported “currently smoking.” Anthropometric measurements such as height (cm) and weight (kg) were obtained through home-based screening during interviews. Body mass index (BMI) was calculated by dividing the weight with height in metres squared. Three blood pressure (BP) readings were taken using the Omron HEM 7120 E Blood Pressure Monitor M2 Basic Digital Intellisense following the standard STEPwise guideline [18]. However, only the second and third BP readings were averaged and used in the data analysis, as the first reading may overestimate the mean BP [19].

The classic FRS was previously validated in Malaysia by Ng and Chia [20]. Meanwhile, Su et.al [9] predicted and compared the 10-year CVD risk among low-income urban dwellers in Metropolitan Kuala Lumpur using both classic and modified FRS, in which both models reported similar findings. We followed the steps proposed by D'Agostino et.al in the prediction of the CVD risk. First, the FRS point from each category was identified and summed up. Then, the total FRS points obtained were converted into 10-year CVD risk, classified as low (≤6%), moderate (7 to 20%), and high (>20%) [9, 17].

### 2.4. Demographic and Socioeconomic of Respondents

The demographic variables included were age, gender, ethnicity (Malay; Chinese; Indian; Aborigine; and others), and marital status (never married; married; separated/divorced; widowed/widower; and others). Socioeconomic variables included were income (below RM 1000; RM1000–RM1999; RM2000–RM2999; and RM3000 and above), highest education level attained (no formal education; primary; secondary; tertiary; and other [e.g., religious school and international school]), and occupation (paid employee; self-employed; homemaker; not working; pensioner; and other). The education status of the respondents was also used as the proxy of the level of health literacy [21, 22].

### 2.5. Dietary and Lifestyle Practice

Lifestyle practice included frequency of eating out and level of total physical activity. Frequency of meals eaten outside acted as a proxy for healthy eating habit [23, 24]. The level and intensity of physical activity were measured by the validated Malay version of the Global Physical Activity Questionnaire (GPAQ) by WHO [25]. The categorisation of the level of total physical activity was low, moderate, and high according to the GPAQ guideline [25].

### 2.6. Statistical Analysis

Data were analysed using IBM SPSS version 24. Descriptive statistics presented the characteristics of the respondents and variables used in deriving FRS scores. Chi-square tests were conducted to examine the association between 10-year CVD risks with the selected independent variables. Multiple linear regression outlined the association between the 10-year CVD point scores with the demographic and socioeconomic variables and lifestyle behaviour.

### 2.7. Ethics Approval

The study was approved by the Monash University Human Research Ethics Committee (MUHREC) (Project ID: 13142). The respondents were given an explanatory sheet and a consent form by the data collectors (DCs) from SEACO during the face-to-face interviews. The DCs conducted the interviews and performed home-based screening after the respondents agreed and signed the consent form.

## 3. Results

A total of 11,897 respondents without a history of CVDs were included in this study.  Table 1 presents the demographic, socioeconomic, and dietary and lifestyle practice data of the respondents. About 35% of the respondents were aged 60 and above. Approximately 57% were female. The majority of the respondents were Malay (63.4%), follow by Chinese (25.1%) and Indian (9.6%). Most of the respondents (88.6%) had at least primary or secondary education. About 53% of the respondents earned below RM1000 on a monthly basis. The majority of the respondents (76%) reported a frequency of eating out of less than 6 meals per week, while about 90% of respondents had a low level of total physical activity.

Table 2 summarises the characteristics of the variables used for constructing the FRS model. The majority of the respondents (87.3%) reported that they did not take antihypertensive medication. About 86% were nonsmokers, and about 90% were nondiabetic patients. The mean age was about 55 years old, and the average of the SBP reading was 132.6 mm/Hg. The mean BMI among the respondents was 26.6 kg/m^2^. The mean predicted CVD risk was 11.29 (95% CI, 11.19 to 11.39).

Table 3 summarises the demographic, socioeconomic, and dietary and lifestyle practice by CVD risk. The results indicate that about 28% and 43% of the respondents were at high and moderate risks of CVD, respectively. Compared to other age groups, respondents aged 70 and above had a higher risk of getting CVD. Male respondents had a high CVD risk. Malay, Chinese, and Aborigine respondents had a high CVD risk compared to the Indian ethnicity. Respondents who were widowed/widower, without formal education, or earned RM1,000–RM1,999 monthly were predicted to have a high CVD risk. The results also show that the risk of getting CVD was quite evenly distributed across all categories for frequency of eating outside per week and level of total physical activity.

Table 4 presents the association between 10-year CVD risk and demographic, socioeconomic, and dietary and lifestyle practice, adjusted for age. This study was aimed at identifying the impact of lifestyle practices with CVD risk prediction among a semirural population; hence, the presentation of two independent models are as follows. Model 1 comprised CVD risk and demographic and socioeconomic variables; while model 2 comprised model 1 in addition to physical activity and dietary practice. By presenting the two models, this study distinguishes the effect of demographic and socioeconomic status and lifestyle practices on CVD risk prediction among the respondents. In model 1, female respondents had lower 10-year CVD risk points (*b* = 1.456, 95% CI, -1.636 to -1.277) as compared to males. Chinese (*b* = −0.765, 95% CI, -0.918 to -0.613) and Indian (*b* = −0.251, 95% CI, -0.471 to -0.031) respondents had lower 10-year CVD risk points as compared to Malay respondents, while the Aborigine group (*b* = 1.797, 95% CI, 1.183 to 2.410) had higher CVD risk points compared to Malay respondents. Only respondents that were widowed/widower had higher CVD risk as compared to never married respondents (*b* = 0.831, 95% CI, 0.456 to 1.207). Respondents that studied primary/secondary level (*b* = −0.875, 95% CI, -1.249 to -0.501), tertiary level (*b* = −1.088, 95% CI, -1.572 to -0.603), and other types of schools (*b* = −0.714, 95% CI, -1.220 to -0.207) had a lower CVD risk as compared to those who had no formal education. Respondents who earned more than RM1,000 monthly had a lower CVD risk as compared to those who earned less than RM1,000 per month. In terms of occupation status, the CVD risk points were higher among the self-employed (*b* = 0.218, 95% CI, 0.017 to 0.418), homemakers (*b* = 0.459, 95% CI, -0.245 to 0.672), those who reported not working (*b* = 0.757, 95% CI, 0.499 to 1.016), and other unspecified occupations (*b* = 0.455, 95% CI, 0.169 to 0.742), as compared to paid employees.

In model 2, those who reported eating solely at home (*b* = −0.342, 95% CI, -0.560 to -0.124) or eating out less frequently (1 to 5 meals per week) (*b* = −0.574, 95% CI, -0.781 to -0.367) had lower predicted CVD risk points as compared to those who ate out 11 times or more per week. There was no association between physical activities with CVD risk prediction. The findings on distal factors are similar to model 1.

## 4. Discussion

The prevalence of predicted CVD risk among the respondents was 28.9%, 42.9%, and 28.2% for low, moderate, and high CVD risk, respectively. The prevalence of CVD risk was lower as compared to a study done in Kuala Langat (a semirural area) in 1993, wherein 55.8% of males and 15.1% of females reported to have a high CVD risk [6]. In contrast, our study shows that 47.2% of males and 13.6% of females had a high CVD risk. However, the prevalence of CVD risk in our study was higher as compared to other past studies in the context of Malaysia. Su et. al. [9] reported that there were 21.8% and 38.9% of respondents who had a high and moderate CVD risk, respectively, among urban dwellers in Kuala Lumpur in 2012. The Prospective Urban Rural Epidemiology (PURE) project conducted in 2008 reported that the prevalence of high CVD risk was only 16% [11]. Ahmad et. al. [26] utilized the NHMS data from 2006 until 2015 to determine the prevalence of CVD risk among Malaysians by using the WHO/ISH risk prediction chart. The authors discovered that the prevalence of high CVD risk (>40%) increased among female respondents aged 70 to 79 with time (11.1% in 2006 to 15.3% in 2015) [26].

The current study shows that there was an association between demographic, socioeconomic, and dietary and lifestyle practice with CVD risk. Older respondents had a higher CVD risk, which is an inevitable event as a process of ageing [26–28]. Older individuals encountered more health issues and sickness such as hypertension [28] and diabetes [27], which are CVD risk factors. Male respondents were found to have a higher CVD risk compared to females. This finding is consistent with the past studies conducted in Malaysia [5, 6, 9]. Meanwhile, Malay, Chinese, and Aborigine respondents had a higher CVD risk compared to the Indian ethnicity. This finding is contrary to the previous studies in Malaysia, where only Malays had a higher predicted CVD risk as compared to other ethnic groups [5, 9]. Meanwhile, Amiri et al. [27] discovered that Indians had a lower risk of having more than one CVD risk factor, while another study showed that Indian males aged 45 and above had higher odds of having more than three CVD risk factors due to dietary and lifestyle practices as well as genetic factors [14]. Moreover, respondents who were widowed/widower were reported to have a higher CVD risk. The findings was consistent with the previous study done in Kuala Lumpur, where married individuals and widows/widowers had a higher CVD risk [9].

In this study, respondents without a formal education had a higher prevalence of CVD risks. Previous studies have shown that low education attainment is one of the contributing factors to CVD risk factors [11, 14]. Individuals with lower education attainment tend to have a lower monthly income [29]. Therefore, respondents who earned RM1,000–RM1,999 monthly in this study had higher prevalence of CVD risk. This is because individuals with a low monthly income will have limited access to health services and encounter a financial burden in obtaining medical support [29]. Subsequently, they tend to lower their own concerns and perceptions on their health conditions [29]. In our study, respondents who were not working or worked as other unspecified occupation had a higher CVD risk. This finding is contrary to that by Amiri et al. [27], where paid employees had a higher CVD risk. Another study in Malaysia found that homemakers had a higher prevalence of CVD risk, attributable to unhealthy lifestyles and dietary practices [14].

The novel finding in our study is the inclusion of Aborigines in the prediction of 10-year CVD risk. They were found to have a higher CVD risk. Previous studies on 10-year CVD risk among Malaysians mainly focused on major ethnic groups such as Malay, Chinese, and Indian [5, 9, 11], and information on Aborigines and minority communities were often excluded in the research evidence reported. Hence, we would like to highly recommend inclusive NCD health policies and strategies by taking into consideration minority communities and Aborigine groups.

Education attainment of respondents is highly associated with the CVD risk. For instance, respondents with a higher education level (tertiary) had a lower CVD risk as compared to those who had no formal education. This result is consistent with some prior studies [5, 11, 30], who report that those with a lower education level had a higher predicted CVD risk. Individuals with a lower education level had a poorer understanding of health information, or they had a lower health literacy in general [14]. Individual's health behaviour could be affected by their health literacy [21]. Individuals with higher education attainment had a higher level of health literacy, where some information and knowledge on health required the need of an individual to read and understand the content [21]. In addition, some past studies proved that health literacy served as a mediator or pathway by which education affects health [21, 22].

In our study, individuals with a higher income (RM1,000 and above) had a lower predicted CVD risk. A low income will limit the individual access to health services. Therefore, the individual's health condition is often neglected [29]. Our finding shows that respondents that worked jobs other than paid employees had a higher CVD risk, which in line with the study by Su et. al [9]. This might be due to paid employees having a stable source of income and insurance coverage that can enhance the access for health services and thus reduce the prevalence of CVD risks [9]. It is noteworthy that individuals with a lower educational attainment had a lower source of income, resulting to limited access to healthcare services [29]. Hence, the predicted CVD risk was high among those with a low education level, those with unstable jobs or unemployed, and those with a low income.

Many studies emphasized the importance of physical activity [31–33] and nutrition intake [34, 35] in CVD prevention and risk reduction. Our study shows that there is no significant association between physical activity and CVD risk, similar to the study by Yang et al. [36]. However, the finding was contrary to the study done by R. Yadav et. al [37], where two week of a yoga-based lifestyle intervention can reduce the predicted CVD risk score by 11%. Meanwhile, a previous study on civil servants in South-Western Nigeria using the WHO prediction chart showed that those who were physically inactive had a 2.4 times higher risk in developing CVD as compared to those who were physically active [38]. Another study in Korea found that young Korean women (below 40 years old) had a high predicted CVD risk due to an unhealthy lifestyle such as smoking, obesity, or sedentary activity [39]. However, the occurrence of CVD is not only influenced by physical activity; socioeconomic status and dietary habits also play an important role in preventing CVD risk. Hence, the insignificant association of physical activity on 10-year CVD risk in our study does not imply that physical activity is not an important aspect in preventing CVD. Since our data focused on reported physical activity, objective measurements of physical activity should be included in future data collection, and this would provide more robust information.

Respondents who reported eating solely at home or eating out less often (less than 6 meals per week) had a lower CVD risk. This might be due to the food choices practised by Malaysians [40]. According to the Household Expenditure Survey Report 2019, people who reside in rural areas use about a quarter of their income on raw foods and ingredients, while those who live in urban areas only spend 16% of their income on similar products [41]. This indicates that people in rural areas have a higher tendency to eat home-cooked food, which is consistent with the findings of this study. The report found that the foods and goods that were most purchased included rice, chicken, eggs, vegetables, fish, beef, and fruits that are essential and nutritious for the body [41]. Many past studies have proven that diets with a low sugar intake help to prevent CVD [34, 42]. Refined carbohydrates (e.g., white rice and white flour), sweetened beverages, high sodium intake, and saturated fats increased CVD risk [34, 43]. In Malaysia, easily accessible fast food outlets [44] and habit of frequently eating out resulted in an increased trend of consuming unhealthy fast foods which are energy dense and high in fat and high in sodium content [45–47]. Meanwhile, people who eat out tend to choose popular food that is high in fats and sodium such as nasi lemak (rice cooked with coconut milk), pasta, chicken rice, and rice with thick and rich gravies. This food choice could increase the CVD risk among those who frequently eat outside. A past study done in Malaysia also concluded that incorrect and disordered eating patterns among Malaysians was associated with the occurrence of obesity, which was one of the risk factors of CVD [48].

This study found that education played an important role in reducing CVD risk. By improving their socioeconomic status through education [29], people are also able to gain knowledge and understand the concept of healthy eating [14]. Therefore, health education should be introduced through formal and informal curricula. Besides that, encouraging home-cooked meals should be a key message for health promotion. This is also in line with the National Plan of Action for Nutrition of Malaysia III 2016–2025 (NPANM III), where the government emphasized promoting healthy eating and active living [4]. Various strategies such as promoting nutrition activities via mass media and conducing advocacy and awareness on Malaysian Healthy Plate concepts through various activities (campaigns, talks, exhibitions, etc.) were implemented in promoting healthy eating [4]. However, these activities are more focused on urban areas. Healthy eating information and education for the rural population should be considered as a priority area for NCD prevention and control. Apart from the Internet, the government should spread health information through broadcasting, newspapers, campaigns, and other offline outlets. This is because older people face difficulty in finding information online, and this causes a low level of health literacy among elderly [49]. Increasing the level of health literacy among older adults is essential, as they are exposed to more health risks and limited access to digital health information [50]. Besides that, financial aid support for low-income groups should be implemented to reduce the financial burden for seeking medical help among the poor. For instance, “Skim Peduli Kesihatan for the bottom 40% income group” (Healthcare Scheme B40 or PeKa B40) was founded as about 48% of the B40 group aged 40 and above had at least one NCD that was often undiagnosed [51].

The strength of this study is the large population representative sample that consisted of the major and minor ethnic groups in the country. This study reported cumulative CVD risk in 10-year prediction, which is a more pragmatic approach than measuring individual risk factors. However, there are some limitations inherent in this study. The use of a self-reported assessment may lead to measurement and recall bias, where the respondents might underreport or overreport the lifestyle practices, particularly the level of physical activity.

## 5. Conclusions

In summary, there is an association between demographic, socioeconomic, and dietary practice with 10-year CVD risk. CVD was one of the five principal causes of death in Malaysia in 2019 [3]. Accessing and utilizing health information interventions should be age friendly where individuals of different age categories can obtain information easily. Increasing the level of health literacy to adopt healthy eating practices among Malaysians should be the focus of the government, in an attempt to reduce CVD risk. Comparison of calories and fat and sodium content of home-cooked and outside meals should be included in health education materials to promote healthy eating at home. Besides the major ethnic groups, public health interventions should also focus on minority communities. It is essential to raise the public's awareness of healthy diets and lifestyle to reduce the rate of mortality due to CVDs.

## Figures and Tables

**Table 1:** Demographic, socioeconomic, and dietary and lifestyle practice of respondents (*N* = 12,477).

Variable	*n*	%
Age groups		
35 to 39	1,249	10.0
40 to 49	3,036	24.3
50 to 59	3,871	31.0
60 to 69	2,717	21.8
70 and above	1,604	12.9
Gender		
Male	5,425	43.5
Female	7,052	56.5
Race		
Malay	7,916	63.4
Chinese	3,129	25.1
Indian	1,197	9.6
Aborigine	136	1.1
Other	99	0.8
Marital status		
Never married	561	4.5
Married	10,129	81.4
Separated/divorced	249	2.0
Widowed/widower	1,460	11.7
Other	78	0.4
Education		
No formal education	413	3.4
Primary/secondary	10,827	88.6
Tertiary	570	4.7
Other	407	3.3
Monthly income^†^		
Below RM1000	6,548	52.5
RM1000–RM1999	3,357	26.9
RM2000–RM2999	1,370	11.0
RM3000 and above	1,202	9.6
Occupation		
Paid employee	3,487	27.9
Self-employed	2,277	18.2
Homemaker	4,299	34.5
Not working	1,487	11.9
Pensioners	883	7.1
Other	53	0.4
Frequency of meals taken outside per week		
0 meal	4,400	35.3
1–5 meals	5,070	40.7
6–10 meals	1,428	11.5
11 meals and above	1,558	12.5
Level of total physical activity		
Low	11,121	89.1
Moderate	649	5.2
High	707	5.7

Notes: ^†^USD $1 is equivalent to RM3.00 (13 May 2013).

**Table 2:** Characteristics of variable in FRS model (*N* = 12,477).

Variable	*n*		%	
On antihypertensive meds				
No	10,893		87.3	
Yes	1,583		12.7	
Smoking status				
No	10,620		85.7	
Yes	1,773		14.3	
Diabetes status				
No	11,183		89.6	
Yes	1,294		10.4	
	Mean	S.D.	Min	Max
Age (years)	55.2	11.8	35.0	98.0
Systolic BP	132.6	19.8	56.0	269.5
BMI	26.6	5.3	7.4	66.2
FRS points	11.3	5.6	-1.0	29.0
CVD risk (%)	13.7	9.8	1.0	30.0

**Table 3:** Demographic, socioeconomic, and dietary and lifestyle practice by CVD risk (*N* = 11,897).

Variables	CVD risk					
	Low (*n* = 3,439)		Moderate (*n* = 5,099)		High (*n* = 3,359)	
	*n*	%	*n*	%	*n*	%
Age groups	*χ* ^2^ = 5903.081∗∗∗					
35 to 39	1,035	87.5	170	14.1	2	0.2
40 to 49	1,557	52.9	1,225	41.6	163	5.5
50 to 59	703	18.9	2,194	58.9	828	22.2
60 to 69	144	5.6	1,116	43.1	1,331	51.4
70 and above	0	0.0	394	27.6	1,035	72.4
Gender	*χ* ^2^ = 2222.545∗∗∗					
Male	551	10.6	2,184	42.2	2,442	47.2
Female	2,888	43.0	2,915	43.4	917	13.6
Ethnicity	*χ* ^2^ = 64.773∗∗∗					
Malay	2,131	28.4	3,252	43.3	2,123	28.3
Chinese	804	26.8	1,298	43.2	900	30.0
Indian	425	36.6	456	39.2	281	24.2
Aborigine	33	24.6	60	44.8	41	30.6
Other	46	49.5	33	35.5	14	15.1
Marital status	*χ* ^2^ = 344.095∗∗∗					
Never married	220	40.9	230	42.8	88	16.4
Married	2,932	30.4	4,122	42.4	2,667	27.4
Separated/divorced	100	41.8	106	44.4	33	13.8
Widowed/widower	150	11.3	619	46.5	561	42.2
Other	23	52.3	14	31.8	7	15.9
Education	*χ* ^2^ = 168.535∗∗∗					
No formal education	45	12.2	158	42.7	167	45.1
Primary/secondary	3,044	29.3	4,442	42.8	2,903	27.9
Tertiary	239	43.6	213	38.9	96	17.5
Other	60	16.3	176	48.0	131	35.7
Monthly income^†^	*χ* ^2^ = 107.428∗∗∗					
Below RM1,000	1,907	30.9	2,638	42.8	1,618	26.3
RM1,000–RM1,999	774	23.9	1,342	41.5	1,117	34.5
RM2,000–RM2,999	401	30.1	583	43.8	348	26.1
RM3,000 and above	357	30.5	536	45.9	276	23.6
Occupation	*χ* ^2^ = 1978.565∗∗∗					
Paid employee	1,200	35.5	1,546	45.8	630	18.7
Self-employed	362	16.5	875	40.0	952	43.5
Homemaker	1,675	40.5	1,917	46.3	547	13.2
Not working	166	12.4	488	36.5	682	51.0
Others	36	4.2	273	31.9	548	63.9
Frequency of meals taken outside per week	*χ* ^2^ = 13.028∗					
0 meal	1,149	28.1	1,731	42.4	1,207	29.5
1–5 meals	1,473	30.0	2,126	43.4	1,304	26.6
6–10 meals	391	28.6	574	42.1	400	29.3
11 meals and above	419	27.5	661	43.4	443	29.1
Level of total physical activity	*χ* ^2^ = 11.576∗					
Low	3,086	29.2	4,532	42.8	2,962	28.0
Moderate	181	28.8	246	39.2	201	32.0
High	172	25.0	321	46.6	196	28.4

Notes: ^†^USD $1 is equivalent to RM3.00 (13 May 2013).

**Table 4:** Regression analysis of Framingham point scores (*N* = 11,897).

Variables	Model 1		Model 2	
	Regression coefficient (95% CI)	S.E.	Regression coefficient (95% CI)	S.E.
Gender				
Male	REF		REF	
Female	-1.456 (-1.636, -1.277)^∗∗∗^	0.092	-1.442 (-1.622, -1.263)^∗∗∗^	0.092
Ethnicity				
Malay	REF		REF	
Chinese	-0.765 (-0.918, -0.613)^∗∗∗^	0.078	-0.859 (-1.015, -0.702)^∗∗∗^	0.080
Indian	-0.251 (-0.471, -0.031)^∗^	0.112	-0.245 (-0.466, -0.025)^∗^	0.113
Aborigine	1.797 (1.183, 2.410)^∗∗∗^	0.313	1.696 (1.077, 2.315)^∗∗∗^	0.316
Others	-0.042 (-0.460, 0.677)	0.367	-0.118 (-0.836, 0.600)	0.366
Marital status				
Never married	REF		REF	
Married	0.191 (-0.120, 0.501)	0.159	0.230 (-0.082, 0.541)	0.159
Separated/divorced	-0.035 (-0.574, 0.503)	0.275	0.001 (-0.537, 0.539)	0.274
Widowed/widower	0.831 (0.456, 1.207)^∗∗∗^	0.192	0.849 (0.473, 1.224)^∗∗∗^	0.191
Others	-0.725 (-1.796, 0.346)	0.546	-0.606 (-1.677, 0.464)	0.546
Education				
No formal education	REF		REF	
Primary/secondary	-0.875 (-1.249, -0.501)^∗∗∗^	0.191	-0.844 (-1.219, -0.469)^∗∗∗^	0.191
Tertiary	-1.088 (-1.572, -0.603)^∗∗∗^	0.247	-1.012(-1.497, -0.527)^∗∗∗^	0.247
Others	-0.714 (-1.220, -0.207)^∗∗^	0.258	-0.661 (-1.167, -0.155)^∗^	0.258
Monthly income^†^				
Below RM1000	REF		REF	
RM1000–RM1999	-0.294 (-0.458, -0.130)^∗∗∗^	0.084	-0.322 (-0.487, -0.157)^∗∗∗^	0.084
RM2000–RM2999	-0.615 (-0.841, -0.389)^∗∗∗^	0.115	-0.664 (-0.892, -0.436)^∗∗∗^	0.116
RM3000 and above	-0.695 (-0.941, -0.450)^∗∗∗^	0.125	-0.796 (-1.047, -0.544)^∗∗∗^	0.128
Occupation				
Paid employee	REF		REF	
Self-employed	0.218 (0.017, 0.418)^∗^	0.102	0.245(0.044, 0.446)^∗^	0.103
Homemaker	0.459 (0.245, 0.672)^∗∗∗^	0.109	0.479 (0.265, 0.692)^∗∗∗^	0.109
Not working	0.757 (0.499, 1.016)^∗∗∗^	0.132	0.790 (0.532, 1.048)^∗∗∗^	0.132
Others	0.455 (0.169, 0.742)^∗∗^	0.146	0.472 (0.186, 0.759)^∗∗^	0.146
Frequency of meals taken outside per week				
11 meals and above			REF	
6–10 meals			-0.176 (-0.435, 0.083)	0.132
1–5 meals			-0.574 (-0.781, 0.367)^∗∗∗^	0.105
0 meal			-0.342 (-0.560, -0.124)^∗∗^	0.111
Level of total physical activity				
High			REF	
Moderate			0.017 (-0.361, 0.396)	0.193
Low			-0.272 (-0.547, 0.003)	0.140
Constant	4.595 (4.070, 5.121)^∗∗∗^	0.268	5.167 (4.559, 5.775)^∗∗∗^	0.310
Observations	11,653		11,637	
Adjusted *R*-squared	0.614		0.616	

Notes: Abbreviation: Reference: REF; *t*-test significance: ^∗∗∗^*p* < 0.001, ^∗∗^*p* < 0.01, and ^∗^*p* < 0.05; values in parentheses are 95% confidence intervals; ^†^USD $1 is equivalent to RM3.00 (13 May 2013).

## Data Availability

Data requestors will need to fill in an online application form from the SEACO website (https://www.monash.edu.my/seaco/research-and-training/how-to-collaborate-with-seaco). All the application will go through the SEACO Review Committee.
